# Physical Activity and the Impact of Continued Exercise on Health-Related Quality of Life Prior to and during Pregnancy: A German Cohort Study

**DOI:** 10.3390/healthcare11152143

**Published:** 2023-07-27

**Authors:** Mariz Kasoha, Amr Hamza, Ayse Leube, Erich-Franz Solomayer, Jochen Frenzel, Roxana Schwab, Romina Marina Sima, Bashar Haj Hamoud

**Affiliations:** 1 Department of Gynecology, Obstetrics and Reproductive Medicine, University Medical School of Saarland, 66421 Homburg, Saarland, Germany; dramrh@gmail.com (A.H.); ayse.leube@web.de (A.L.); erich.solomayer@uks.eu (E.-F.S.); bashar.hajhamoud@uks.eu (B.H.H.); 2 Kantonsspital Baden, Im Ergel 1, 5404 Baden, Switzerland; 3 Frauenarztpraxis, Berliner Promenade 15, 66111 Saarbrücken, Germany; info@dr-jochen-frenzel.de; 4 Department of Gynecology and Obstetrics, University Medical Center Mainz, Langenbeckstraße 1, 55131 Mainz, Germany; roxana.schwab@unimedizin-mainz.de; 5 Department of Obstetrics and Gynecology, ‘Carol Davila’ University of Medicine and Pharmacy, 020021 Bucharest, Romania

**Keywords:** physical activity, pregnancy, consistency, HRQoL, moderate, strenuous, trimesters

## Abstract

The goal of this study was to examine how regular physical activity before and during pregnancy affected life quality throughout pregnancy. Between July 2020 and May 2021, 218 pregnant women were recruited from 11 outpatient clinics for this survey. Data were collected prospectively in a panel format beginning with the 10th gestational week over a 20-week period. Prior to pregnancy, a previous time point was also defined. The International Physical Activity Questionnaire, the EQ-5D-3L questionnaire, and the EQ-VAS questionnaire were used to collect data on the duration and intensity of daily physical exercises, as well as to assess health-related quality of life and self-estimated health status. The final survey included data from 113 women. During pregnancy, physical activity decreased dramatically. The duration of strenuous activities, but not moderate activities, was significantly reduced. Continuous physical activity independently predicted higher life quality scores at all points of assessment. Cases who participated in moderate and strenuous activities on a regular basis had higher self-estimated health status scores than cases who only participated in moderate activity. Instead of focusing solely on specific types of physical activity, we believe that strategies for motivating all pregnant women to be constantly active should be developed.

## 1. Introduction

Studies show that regular exercise improves maternal health. It enhances maternal cardiovascular function, reduces musculoskeletal discomfort, diminishes weight gain, and decreases the risk of preterm birth, gestational diabetes, and gestational hypertension [1].

Physical inactivity has been identified as the fourth leading risk factor for global early mortality. As a result, national and international guidelines recommend physical activity prior to and following pregnancy [2]. Despite the evident benefit, numerous studies showed that women reduced or even stopped their physical activity after the onset of pregnancy [3,4,5,6,7,8,9]. Furthermore, sedentary activities increased throughout pregnancy [10,11]. Few women performed the bare minimum of exercise recommendation before or during gestation [12]. In a Brazilian study, 14.8% and 12.9% of the women reported engaging in some type of physical activity prior to and during pregnancy, respectively, with 4.3% active throughout the pregnancy [3]. Most studies found that pregnant women increased their physical activity in the second trimester and decreased it in the third [10,13]. During all three trimesters, the majority of the women walked [3,6].

Using health-related quality of life (HRQoL) scores to assess pregnancy-related symptoms, physical exercise has shown a healthy quality improvement, as in blood pressure [14], depression [15,16], and musculoskeletal-related symptoms [17]. Krzepota et al. reported a significant increase in the life quality level in polish pregnant women with increased physical activity in the second and third trimesters [11]. Similarly, Mourady et al. documented this observation in their study at three measurement time points [18]. Contrarily, Tendais et al. showed that physical and mental components are influenced differently by pregnancy course, regardless of physical activity status [19].

The correlation between physical activity and quality of life, examined in the aforementioned studies, is limited to specific measurement time points, e.g., specific week of gestation (WG) or specific trimester. Therefore, it is not possible to draw objective conclusions about the impact of physical activity on HRQoL levels throughout pregnancy. Consequently, we designed a study to assess the impact of physical activity before and during gestation at different gestational weeks (GW) (10th, 20th, and 30th GW). To the best of our knowledge, no such data have been found in the literature in Germany. 

## 2. Materials and Methods

### 2.1. Research Goals

The aim of this study is to collect data on the physical and athletic activity and quality of life of pregnant women before pregnancy and in the weeks 10, 20, and 30 of pregnancy in order to answer the following central research questions:

1. How physically and athletically active are pregnant women during pregnancy?

2. Is there a correlation between physical activity and health-related quality of life in pregnant women?

### 2.2. Study Design and Participants

This survey was conducted between July 2020 and May 2021. Pregnant women were recruited in eleven outpatient clinics. The data were collected prospectively in a panel format over 20-week period. We defined one retrospective time point before the pregnancy (T1) and three prospective measurement time points during pregnancy (T2: 10th GW, T3: 20th GW, and T4: 30th GW). The pre-pregnancy data were collected from participants at the same time as the first interview (T2) to determine whether certain sociodemographic characteristics influence the discontinuation or continuation of physical activity during pregnancy.

Inclusion criteria were pregnant women ≥18 years of age with a voluntary consent, gestational age ≤10th GW, and basic ability to participate in sports. Exclusion criteria were a physician’s prohibition of sports or strenuous physical activity during pregnancy and all pregnant women whose pregnancies were ended before the 30th week of gestation.

### 2.3. Data Collection

For comparison, the questionnaires were identical at each measurement time point. We used a short form and German version of the International Physical Activity Questionnaire (IPAQ) [20]. We performed the questionnaires as paper–pencil surveys in the physicians’ offices and anonymized the questionnaires by an individual code. 

The survey included the questions about duration and intensity of various daily physical exercises such as walking, jogging, swimming, bicycling, inline skating, yoga, gymnastics, aerobics, fitness centre training, and others. We defined moderate activity as a physical effort that require little more than normal breathing. Strenuous activity was defined as a physical effort that require significantly more breathing than normal.

To assess HRQoL, we used a registered simplified three-answer option EQ-5D-3L questionnaire. The EQ-5D questionnaire is a validated tool to measure health-related quality of life in five dimensions (mobility, self-care, general activities, pain/physical discomfort, and anxiety/depression) [20]. Therefore, we registered the study at the Euroquol. We also included a vertical visual analogue scale (EQ-VAS). In which participants were asked to rate their health status on a scale of 0 to 100, with zero representing the worst possible and 100 representing the best possible health status.

Data on age, BMI, number of children, and type of health insurance were also recorded. In addition, in an open-ended response, we asked participants to report their occupation. The responses were classified in three groups as academic, medium level of education, and housewives or unskilled.

### 2.4. Evaluation

Due to the importance of the impact of continued physical activity on the respective dependent variable (e.g., quality of life), we reported the activity status and intensity, as well as the types of sports practiced at each measurement time point. Another variable investigated was the persistence of physical activity during pregnancy, as shown in Table 1.

**Figure 1:**
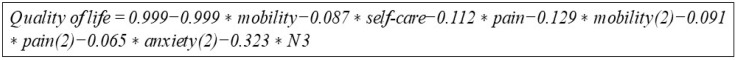
Greiner equation. The number 2 in brackets (2) will only be included if the participant indicated the highest grade on the evaluation scale for the respective problem. “N3“was so named by Greiner et al. It indicated that any of the three given problems was referred to as being extreme (grade 3) [20].

Data for the EQ-5D were calculated using Greiner et al. validated model [20]. The resulting variable is interval-scaled and polarized, with high values representing high quality of life and low values representing low quality of life. The maximum value of 1.0 is only theoretically possible, as demonstrated by the equation displayed in Figure 1.

### 2.5. Statistical Analysis

IBM SPSS version 26 (IBM Corp., Armonk, NY, USA) was used to analyse the data. A test power calculation was performed for the group comparisons using the program G*Power. The statistical power of the T1, T2, T3, and T4 groups was 0.401, 0.888, 0.688, and 0.885 for SEHS scores and 0.275, 0.885, 0.887, and 0.973 for HRQol scores, respectively. The parametric tests used here were based on the group size of more than 30 cases. Numerical data were presented as average and standard deviation (STD) and analysed using the t-Student Test. Categorical data were presented as numbers and percentages and analysed using the Chi-squared test. To investigate changes over time within one group, the analysis of variance (ANOVA) method was used. Multivariate logistic regression analysis was used to test the impact of physical activity engagement and consistency in predicting HRQoL and self-estimated health scores at each measurement point. The level of statistical significance of <0.05 was considered significant.

## 3. Results

**Figure 2:**
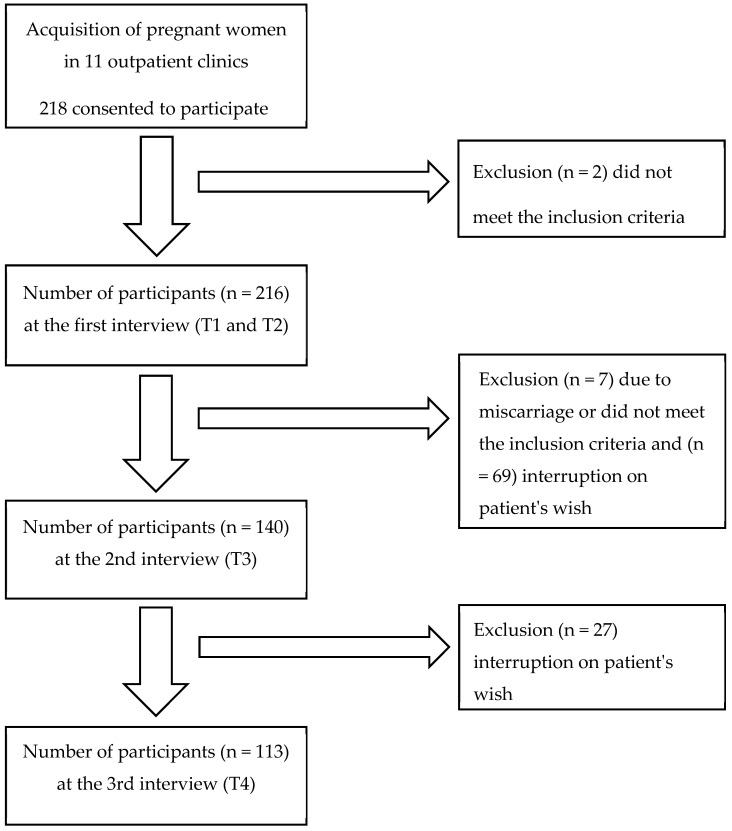
Flowchart of included study cases. Inclusion criteria were pregnant women ≥18 years of age with a voluntary consent, gestational age ≤10th GW, and basic ability to participate in sports. Exclusion criteria were a physician’s prohibition of sports or strenuous physical activity during pregnancy and all pregnant women whose pregnancies were ended before the 30th week of gestation.

This survey included 218 participants from 11 outpatient clinics. For the T1 and T2 measurement time points, 216 participants provided data. The remaining two participants were excluded because they did not meet the inclusion criteria. At the T3 measurement time point, data from 140 participants were available after excluding seven patients due to exclusion criteria, with 69 participants discontinuing on their own terms. At the last survey, T4, 27 women dropped out, leaving data from 113 women for final analysis. Figure 2 represents a flowchart of study cases.

### 3.1. Study Population

The mean age of the participants was 30.95 ± 5.4 years. Data showed that 20% (41/216) of study cases had two or more children, 32% (70/216) had only one child, and 48% (105/216) had none. Seven women had multiple pregnancies. Due to this number, no subgroup analysis was possible. Approximately 90% of participants had public insurance, while 10% had private health insurance. In addition, we found that 22.2% of the study participants had an academic occupation, 57.9% had an intermediate occupation, 6% were unskilled, and 4.6% were housewives. No assignment was possible for the remaining 9.3% (Table 2).

### 3.2. Baseline Population Data Recorded at Different Intervals

Different measurements that were recorded at study intervals are presented in Table 3. The best self-estimated health status and life quality scores were recorded at T1. With the onset of pregnancy, the proportion of cases with no practiced physical activity were increased by 16%, 13%, and 26% at T2, T3, and T4, respectively. In addition, we observed that the number of days per week and duration per day of moderate and/or strenuous activities were decreased throughout the gestation compared with the data before pregnancy. Results of walking activity showed the same scenario. Participants, on the other hand, had a longer duration of sedentary activities during pregnancy than at T1. The results showed a wide span of data.

Data on the proportion of women practicing different types of sports during pregnancy are presented in Table 4. We found that most types of practiced sports were reduced throughout the pregnancy period except for walking which increased from 29.6% at T1 to 32.7% at T4. Moreover, compared with T1, the proportion of women who practiced yoga decreased at T2 and T3 and increased again at T4.

The proportions of women with activity over their pregnancies and trimester-specific participation percentage are summarized in Table 5. Prior to pregnancy, approximately [79% (170/215)] of all participants engaged in moderate physical activity at least one day per week. This proportion decreased at the beginning of pregnancy [70% (149/214)] and then increased slightly toward the second trimester [73% (99/136)]. At T4, only [59% (65/111)] of women engaged in moderate physical activity. The same scenario was observed in participants engaged in strenuous physical activity [at T1: 65% (140/216), at T2: 36% (78/216), at T3: 42% (59/140), and at T4: 35% (38/110)]. Women who engaged in continuous activity, on the other hand, showed a gradually decreasing proportion of participation in both moderate and strenuous activities. Strenuous physical activity had the most distinct reduction. In addition, the proportion of women who participated in at least one sport decreased by 26% at the start of pregnancy and slightly decreased with the advanced gestation age. This proportion decreased by 13% and 22% in women who continued practicing activities at T3 and T4 compared with T2.

### 3.3. Duration of Strenuous and Moderate Activities, Walking and Sitting throughout Pregnancy Period

**Figure 3:**
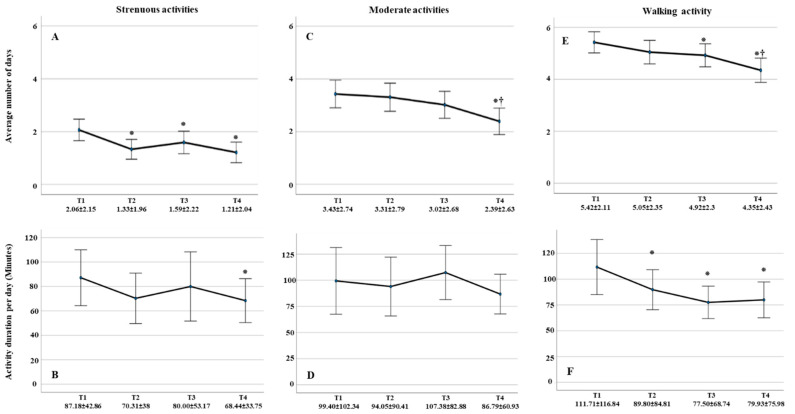
Number of days and duration per day in minutes for strenuous-, moderate-, and walking activities throughout the pregnancy. (**A**) Average number of days of strenuous activities engagement reduced significantly by pregnancy onset. (**B**) Strenuous activities duration per day reduced significantly at T4 compared with T1. (**C**) Average number of days of moderate activities engagement reduced significantly at T4 compared with T1. (**D**) Duration of practicing moderate activities per day showed no significant differences between all measurement points. (**E**) Average number of days practicing walking reduced at T3 and T4 compared with T1. (**F**) Walking duration per day reduced significantly throughout gestation. Data displayed as average ± STD. ⁕ significancy compared with T1. † Significancy compared with previous measurements.

These data were calculated in cases, which participated in all four measurement points (N = 113). As shown in Figure 3A, there was a significant decrease in strenuous activity at T2, T3, and T4 when compared to T1 (*p* = 0.001, *p* = 0.037, *p* **<** 0.001, respectively) and remained unchanged until the third trimester (T2 vs. T3: *p* = 0.242; T2 vs. T4: *p* = 0.573; T3 vs. T4: *p* = 0.083). Compared to T1, there was a statistically significant decrease of the strenuous activity per day in minutes in the last trimester of gestation T4 (*p* = 0.048) (Figure 3B). 

Number of days per week of moderate activity decreased significantly at T4 compared with T1, T2, and T3 (*p* < 0.001, *p* = 0.001 and *p* = 0.003, respectively) (Figure 3C). However, the average duration per day remained constant throughout gestation (Figure 3D). 

There was an insignificant decrease in walking days with the onset of pregnancy (*p* = 0.055), which becomes significant in the second and third trimesters (*p* = 0.041, *p* < 0.001) (Figure 3E). In addition, the average walking duration per day decreased significantly with advanced gestation compared with duration before pregnancy onset (T2: *p* = 0.047, T3: *p* = 0.010, and T4: *p* = 0.012) but becomes insignificant with progressive pregnancy (T2 vs. T3: *p* = 0.112, T2 vs. T4: *p* = 0.231, and T3 vs. T4: *p* = 0.691) (Figure 3F). Women tended to walk around 20 to 30 min more before pregnancy than during pregnancy. 

On the other hand, hours of sitting per day increased at T2 (7.70 ± 3.69) and T4 (7.64 ± 3.96) compared with T1 (6.70 ± 3.22) (*p* = 0.002 and *p* = 0.025, respectively). Duration at T3 (7.13 ± 3.86) showed no significant difference compared with T1 (*p* = 0.169). There was no significant change with progressive pregnancy (T2 vs. T3: *p* = 0.062, T2 vs. T4: *p* = 0.874, and T3 vs. T4: *p* = 0.105).

### 3.4. The Correlation of Discontinuation of Sporting Activity with Different Clinical Characteristics

We had 113 cases who continued to participate in this study at the T4 measurement point. A total of 40 cases (35%) had continuous physical activity throughout the pregnancy, while 73 (75%) had their physical activity interrupted at one measurement point.

At T2, our findings revealed that discontinuation of the sporting activity was correlated with maternal age and BMI. Women over the age of 35 are more likely to continue physical activity than women under the age of 35 [Chi2 (df = 1) = 3.452, *p* = 0.037], and women who stopped exercising had a lower BMI than those who continued [Average STD: 24.335.85 vs. 26.235.11; Chi2 (df = 182) = 2.266, *p* = 0.025]. 5. HRQoL and Self-Estimated Health Status Scores and Their Correlation with Physical Activity throughout the Pregnancy.

Pre-pregnancy HRQoL scores were significantly higher than at any other time point during gestation (all *p* < 0.001). From T2 to T3, there was a non-significant increase in quality of life, followed by a significant decrease from T3 to T4 (*p* **<** 0.001). Furthermore, women who exercised continuously during pregnancy had moderately higher HRQoL scores than those who did not exercise continuously. When comparing women’s self-assessed health status scores, a similar scenario of results was observed. Scores during pregnancy were consistently higher in the active group than in the inactive group. Moreover, we found that the reduction in both scores during pregnancy was less pronounced in the active group compared to the inactive group. At T4, the reduction in HRQoL scores and women’s self-assessed health status scores in the inactive group was 9% and 12% higher than in the active group, respectively (Table 6).

Then, using multivariate logistic regression analysis, we examined the impact of physical activity in predicting HRQoL scores at each measurement point when adjusted for age, BMI, having children, occupation, and type of health insurance. This analysis was performed for all cases included in the study. Our findings revealed that engaging in physical activity has a positive impact on HRQoL scores across all measurement points. The same analysis was used to examine the effect of physical activity consistency on HRQoL scores in participants who remained active until T4. Throughout the study period, we found that physical activity consistency has an independent positive impact on HRQoL scores as well as self-estimated health status scores (Table 7).

**Figure 4:**
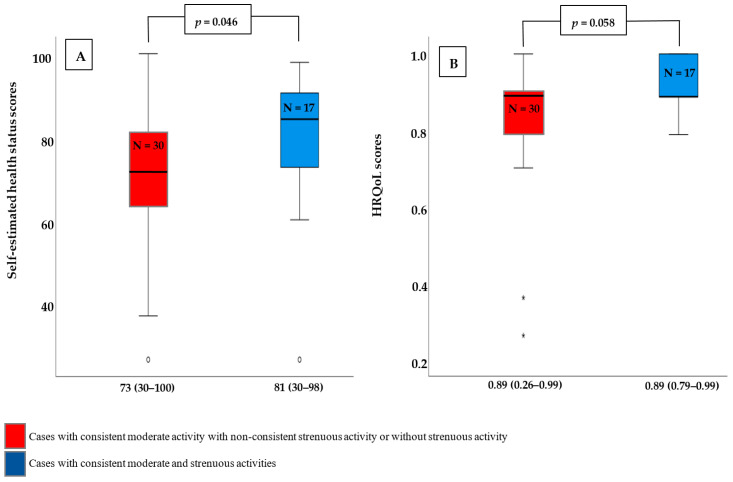
Self-estimated health scores and HROoL scores according to the type of consistent physical activity. (**A**) Self-estimated health scores were significantly higher in those who engaged in consistent moderate- and strenuous activities compared to those who engaged in consistent moderate activity with non-consistent strenuous activity or none at all. (**B**) Cases with consistent moderate- and strenuous activities had higher HRQoL scores than cases with consistent moderate activity but no consistent strenuous activity or no strenuous activity. This difference, however, was not statistically significant. Data displayed as Mean (Range). *p*-value is calculated using Mann–Whitney-U-Test. N: Number of cases.

We divided the cases who continued practicing their physical activities until T4 into two groups to investigate whether the impact of physical activity consistency on self-estimated health scores and/or HROoL scores is affected by the type of activity. The first group included cases with consistent moderate and strenuous activities, while the second group included cases with consistent moderate activity but no consistent strenuous activity. We found that consistent participation in both types of physical activities significantly improves self-estimated health scores and has a tendency to improve HROoL scores (Figure 4).

## 4. Discussion

In our study, we found that strenuous and moderate physical activities decrease with the onset of and throughout pregnancy. Being under the age of 35 and having a lower BMI were factors that contributed to the discontinuation. Consistent exercise contributed to a higher quality of life in our study group. 

Prior to pregnancy, 90% of the participants were physically active (11% in strenuous, 25% in moderate, and 54% in combined moderate and strenuous activities). In the first trimester (T2), this number was reduced to 74% (4% in strenuous, 38% in moderate, and 32% in combined moderate and strenuous activities). Other studies also show a similar reduction of physical or sporting activities with the onset of pregnancy [6,9]. Haakstad et al. reported 19% (vs. 10% in our study) were non-exercisers before pregnancy, 30% in the first trimester, 36% in the second trimester, and 53% in the third trimester. A possible reason for the physical activity reduction during the first trimester is first-trimester complications and discomfort, e.g., nausea [21,22]. Unlike what was expected, the participation increased only to 77% (4% in strenuous, 37% in moderate, and 36% in combined moderate and strenuous activities) in the second trimester. One would expect more engagement in physical activity within the more “comfortable” second trimester. Due to the increasing abdominal girth and more exposure to pregnancy discomfort in the third trimester, the reduction of physical exercise in our study collective was expected. In the 3rd trimester (T4), only 64% remained active (4% in strenuous, 27% in moderate, and 33% in combined moderate and strenuous activities). Different studies, like ours, found that physical activities were lowest during the third trimester [6,10]. One possible explanation is a false fear of harming the foetus while exercising [22,23]. In the setting of an uncomplicated pregnancy, these concerns were not found to be true [24,25,26,27,28]. A further possible cause specific to our study collective could also be the restrictive nature of the Covid pandemic. In general, the era of the Covid pandemic resulted in various psychological problems, which resulted in overdependence on social media and decreased physical activity [29]. Yet, at least 150 min of moderate or 75 min of strenuous activity is recommended per week during pregnancy [30]. Physical inactivity is harmful to pregnant women and contributes significantly to premature mortality worldwide [31]. Therefore, it is recommended to encourage women with physiological pregnancies to engage in aerobic and strength-conditioning exercises throughout gestation. As a result, it is recommended that women with physiological pregnancies engage in aerobic and strength-conditioning exercises throughout their pregnancy [32].

Participating in non-hazardous sports increased with ongoing pregnancy. In our study, collective walking increased from slightly less than 30 percent before pregnancy to about one-third. Practicing Yoga and gymnastics decreased with onset and increased with ongoing gestation. Our findings are in line with the literature [3,6]. These exercises in pregnancy were extensively studied and found to be safe [33]. More hazardous or vigorous physical activity, e.g., cycling or inline skating, decreased steadily in the course of pregnancy. As expected, and in line with the literature, practicing physical exercise in a gym also decreased throughout pregnancy. A possible explanation for the latter one is the onset of the Covid pandemic [29,34]. Vigorous exercise was proven to be associated with a significant decrease in foetal weight. However, it was not associated with an increased rate of foetal growth restriction [35,36,37]. Currently, there is lacking evidence on the effect of vigorous physical activity on pregnancy [33].

In terms of practicing frequency per weak, we discovered that while strenuous activity frequency decreased significantly from the first trimester, walking and moderate activity frequency decreased significantly from the second and third trimesters, respectively. Furthermore, the average duration of moderate physical activity per day remained constant throughout pregnancy, allowing us to conclude that pregnancy was not an impediment or driver to moderate physical activity and that women are perfectly capable of performing moderate physical activities throughout pregnancy as they did before pregnancy. 

Contrary to the literature, at the beginning of pregnancy, we found an association only between the age of the pregnant women (<35 years) and the cessation of physical activity [5]. In addition, we found a negative correlation between increased BMI and the cessation of physical activity. In a more recent study, a more favourable self-rated health before pregnancy onset and a lower BMI was associated with higher levels of physical activity [38]. In a review of 16 studies higher education and income, not having children at home, being white and more active before pregnancy was associated with higher exercise during pregnancy [39]. In our collective, education and the number of pre-existing children were not correlated to abandoning physical activity during gestation. This could be explained by cohort bias.

One of the most important implications of our study is that it investigates not only the impact of physical activity on quality of life but also the effect of consistency of physical activity participation. First, we found that the proportion of women who engage in physical activity, whether moderate or strenuous exercise or participation in a single sport decreased after pregnancy begins. However, when there was consistent exercise, less reduction in practicing strenuous activities was reported by the third trimester. These findings imply that continuous exercise may have a positive impact on exercise intensity. Second, the findings on quality of life highlight the importance of consistent physical activity throughout pregnancy. We found that HRQoL and self-reported health status were lower at all measurement points during pregnancy compared with pre-pregnancy scores. When compared to cases with continuous engagement, this reduction in the third trimester was greater in cases with uncontentious physical activity participation by at least 58% and 39% for HRQoL- and self-estimated health status scores, respectively. Furthermore, physical activity participation and consistency have been shown to independently predict HRQoL- and self-estimated health status scores.

Nevertheless, we found that many women reduced or stopped their physical activities during pregnancy. Our study further shows that it cannot be assumed that low-risk pregnant women will remain physically active with the onset of pregnancy. In different studies, exercise during pregnancy was shown to maintain physical fitness, decrease the risk of many complications, including gestational diabetes, hypertensive diseases, caesarean birth, and operative vaginal birth rates, increased postpartum recovery time, depressive disorders, and body pain, e.g., lumbar and sciatic pain [33,40,41,42,43,44,45,46,47,48,49,50,51,52]. It is not surprising, therefore, that international guidelines exist to encourage pregnant women to participate in physical activity during their pregnancy [30,31,32]. Based on our findings, we recommend women with uncomplicated pregnancies be encouraged to engage in physical exercise throughout gestation [2].

To the best of our knowledge, this is the first study to investigate the physical activity of women living in Germany prior to and during pregnancy. The main strength of this study is the analysis of how pregnant women’s HRQoL changed from prior pregnancy over a 20-week period with three measurement points during pregnancy, allowing us to exclude the effect of complications prior to and during pregnancy. Furthermore, data from such a large sample size might serve to offer utility-based case values in pregnant women at different stages of gestation in the clinic, as well as contribute to health economic research. It should be noted, however, that more closely timed data collection might allow for a more accurate assessment of causal relationships. Further, this survey was conducted anonymously. Therefore, it is acceptable to speculate that participants’ willingness to respond in terms of social desirability had no significant influence on the current data.

## 5. Conclusions

Our study highlighted a possible positive effect of physical activity behaviour in pregnant women on the HRQoL. Yet, the direct effect of physical activity on the quality of life still has to be examined in further studies. However, based on our findings, and in line with international recommendations, all pregnant women should be counselled on the advantages of physical activity early in pregnancy.

## Figures and Tables

**Table 1:** Examples of physical activity persistence during pregnancy.

Case	Before Pregnancy (T1)	10th GW (T2)	20th GW (T3)	30th GW (T4)
1	active	active	active	active
2	active	active	non active	active
3	active	non active	active	active
4	non active	active	non active	non active

Case No.1 is considered to have continuous activity because the patient was active at each measurement time point. Case No.2 is considered to be active prior to pregnancy (T1) and at the 10th GW (T2) but not by T3, indicating that activity is restricted to the period preceding T3. Case No.3 is listed as inactive throughout the pregnancy, and Case No.4 is listed as consistently inactive. What matters is that there was always activity in the respective area prior to the time point in question.

**Table 2:** Demographic features of study cases.

Age (Years) (Average ± STD)		30.95 ± 5.4
Number of children	Non	105 (49%)
	One child	70 (32%)
	≥2	41 (19%)
Occupation	Academic	43 (22%)
	Intermediate occupation	130 (58%)
	Not skilled	13 (6%)
	Housewives	10 (5%)
	Unknown	20 (9%)
Health insurance type	Public	194 (90%)
	Private	22 (10%)

Data displayed as average ± STD and as number and percentage.

**Table 3:** Baseline population data was obtained at different intervals.

	T1	T2	T3	T4
BMI	Not obtained	N = 216	N = 130	N = 107
		25.74 ± 5.77	26.86 ± 5.53	29.53 ± 5.58
Self-estimated health status score	N = 215	N = 216	N = 138	N = 112
	86.80 ± 14.29	74.94 ± 20.02	79.15 ± 16.06	71.09 ± 19.89
Mobility	N = 216	N = 216	N = 140	N = 113
Normal	216 (100%)	186 (86%)	112 (80%)	73 (65%)
Not normal	0 (0%)	30 (14%)	28 (20%)	39 (35%)
Self-care ability	N = 216	N = 216	N = 140	N = 113
Normal	216 (100%)	208 (96%)	140 (100%)	104 (92%)
Limited	0 (0%)	8 (4%)	0 (0%)	9 (8%)
Daily activities	N = 216	N = 216	N = 140	N = 113
Normal	211 (97.5%)	168 (78%)	123 (88%)	79 (70%)
Limited	4 (2%)	43 (20%)	16 (11%)	30 (27%)
Very limited	1 (0.5%)	5 (2%)	1 (1%)	4 (3%)
Pain/physical discomfort	N = 216	N = 216	N = 140	N = 113
None	187 (86.5%)	95 (44%)	81 (58%)	32 (28%)
Moderate	28 (13%)	98 (45%)	52 (37%)	73 (65%)
Strong	1 (0.5%)	23 (11%)	7 (5%)	8 (7%)
Fears/Despondency	N = 216	N = 216	N = 140	N = 113
None	191 (88.5%)	166 (77%)	114 (81%)	89 (79%)
Moderate	24 (11%)	45 (21%)	22 (16%)	20 (18%)
Strong	1 (0.5%)	5 (2%)	4 (3%)	4 (3%)
HRQoL score ^†^	N = 216	N = 216	N = 140	N = 113
	0.98 ± 0.07	0.85 ± 0.22	0.89 ± 0.18	0.82 ± 0.21
Type of practiced activity	N = 216	N = 216	N = 140	N = 113
None	21 (10%)	56 (26%)	32 (23%)	41 (36%)
Strenuous	24 (11%)	9 (4%)	5 (4%)	5 (4%)
Moderate	55 (25%)	82 (38%)	52 (37%)	31 (27%)
Both	116 (54%)	69 (32%)	51 (36%)	36 (33%)
Moderate activities				
Days a week	N = 215	N = 214	N = 136	N = 111
	3.46 ± 2.65	3.01 ± 2.71	2.96 ± 2.61	2.44 ± 2.65
Hours a day	N = 159	N = 143	N = 90	N = 62
	1.72 ± 1.62	1.51 ± 1.29	1.61 ± 1.53	1.30 ± 0.96
Strenuous activities				
Days a week	N = 216	N = 216	N = 140	N = 110
	2.03 ± 2.04	1.10 ± 1.85	1.49 ± 2.17	1.21 ± 2.03
Hours a day	N = 135	N = 76	N = 55	N = 35
	1.54 ± 1.25	1.43 ± 1.31	1.26 ± 0.95	1.08 ± 0.55
Walking				
Days a week	N = 216	N = 215	N = 137	N = 111
	5.31 ± 2.14	4.84 ± 2.40	4.93 ± 2.32	4.32 ± 2.40
Hours a day	N = 195	N = 189	N = 120	N = 96
	1.91 ± 2.02	1.54 ± 1.55	1.24 ± 1.12	1.36 ± 1.41
Sedentary activities				
Hours a day	N = 204	N = 197	N = 131	N = 99
	6.12 ± 3.21	7.36 ± 3.59	7.26 ± 3.63	7.65 ± 4.11

Data displayed as average ± STD for continuous data and as number and percentage for categorical data. N: number of cases. ^†^ Health-related quality of life score is composed of the five dimensions as explained.

**Table 4:** The proportion of women practicing different types of sports during pregnancy.

Type of Sport	T1 (N = 216)	T2 (N = 216)	T3 (N = 140)	T4 (N = 113)
Running	21.3%	3.2%	1.4%	0.9%
Walking	29.6%	31.9%	36.4%	32.7%
Swimming	23.1%	13.0%	9.3%	1.8%
Bicycle riding	30.6%	12.5%	9.3%	5.3%
Inline Skating	5.1%	0.9%	0.7%	0%
Yoga	14.8%	7.4%	12.9%	16.8%
Gymnastics	12.0%	8.3%	11.4%	11.5%
Aerobic	7.9%	4.%	2.9%	0.9%
Gym	20.8%	4.6%	2.1%	0.9%
Extreme sports	3.7%	0%	0%	0%
Others ^†^	25.5%	9.3%	7.1%	4.4%

Data displayed as percentage. N: number of cases. ^†^ Different types of activities such as dancing, riding, climbing, and working in the garden.

**Table 5:** Proportion of women with consistent and inconsistent physical activity throughout the gestation.

Time of Data Collection	Proportion of All Women			Proportion of Women with Continuation of Described Activity		
	Moderate Physical Activities	Strenuous Physical Activities	Participation in One Sport at Least	Moderate Physical Activities	Strenuous Physical Activities	Participation in One Sport at Least
T1	79% (170/215)	65% (140/216)	85% (187/216)			
T2	70% (149/214)	36% (78/216)	59% (127/216)	64% (138/215)	34% (74/216)	57% (123/216)
T3	73% (99/136)	42% (59/140)	58% (81/140)	57% (77/136)	24% (33/140)	44% (61/140)
T4	59% (65/111)	35% (38/110)	51% (58/113)	43% (46/107)	16% (17/108)	35% (40/113)

Data displayed as percentage and number.

**Table 6:** HRQoL- and self-estimated health status scores according to physical activity throughout the pregnancy.

		All Cases (N = 113)	Reduction% Compared with T1	Cases with Continued Physical Activity (N = 39)	Reduction% Compared with T1	Cases with Uncontinued Physical Activity (N = 70)	Reduction% Compared with T1	^†^ *p*
HRQoL scores	T1	0.99 ± 0.05		0.99 ± 0.03		0.98 ± 0.06		0.299
	^Δ^ T2	0.86 ± 0.22	13%	^Δ^ 0.93 ± 0.13	6%	^Δ^ 0.83 ± 0.24	15%	0.019
	^Δ^ T3	0.89 ± 0.18	10%	^Δ^ 0.95 ± 0.07	4%	^Δ^ 0.86 ± 0.21	12%	0.041
	T4	0.82 ± 0.21	17%	0.90 ± 0.09	9%	0.77 ± 0.24	21%	0.003
SEHS scores	^Δ^ T1	88.39 ± 12.5		^Δ^ 90.72 ± 9.57		^Δ^ 87.36 ± 13.96		0.415
	^Δ^ T2	74.5 ± 19.8	15%	^Δ^ 81.39 ± 14.18	11%	^Δ^ 70.80 ± 21.82	18%	0.010
	^Δ^ T3	78.87 ± 16.30	10%	^Δ^ 83.21 ± 15.78	9%	^Δ^ 76.33 ± 16.26	13%	0.014
	T4	71.09 ± 19.88	19%	^Δ^ 78.41 ± 16.90	14%	67.21 ± 20.57	23%	0.003

SEHS: Self-estimated health status. ^†^: *p* is used to compare cases with and without continuous activity. ^Δ^: *p <* 0.05 comparing with later measurement points within the same group. Data displayed as average ± STD. *p* is calculated using ANOVA test.

**Table 7:** Impact of physical activity in predicting HRQoL- and self-estimated health status scores throughout the gestation.

	Time of Data Collection	(B)	Odds Ratio	95% CI (Lower Limit–Upper Limit)	*p*
* Physical activity	T1	0.026	0.136	0.00–0.052	0.050
	T2	0.107	3.593	0.048–0.166	<0.001
	T3	0.091	0.249	0.028–0.154	0.005
	T4	0.139	0.332	0.059–0.219	<0.001
° Physical activity consistency	T2 SEHS HRQoL	10.092 0.117	0.244 0.258	2.338–17.846 0.034–0.201	0.011 0.006
	T3 SEHS HRQoL	7.041 0.090	0.203 0.238	0.194–13.887 0.014–0.166	0.044 0.021
	T4 SEHS HRQoL	10.785 0.118	0.257 0.271	2.686–18.884 0.034–0.202	0.010 0.006

SEHS: Self-estimated health status. Data adjusted for age, BMI, number of children, occupation, and type of health insurance. * The effect of engagement in physical activity on HRQoL scores. ° The effect of physical activity consistency on HRQoL- and SEH scores.

## Data Availability

The data that support the findings of this study are available on request from the corresponding author (M.K.).
